# Cockayne Syndrome: Varied Requirement of Transcription-Coupled Nucleotide Excision Repair for the Removal of Three Structurally Different Adducts from Transcribed DNA

**DOI:** 10.1371/journal.pone.0094405

**Published:** 2014-04-08

**Authors:** Nataliya Kitsera, Karola Gasteiger, Bork Lühnsdorf, Julia Allgayer, Bernd Epe, Thomas Carell, Andriy Khobta

**Affiliations:** 1 Institute of Pharmacy and Biochemistry, Johannes Gutenberg University of Mainz, Mainz, Germany; 2 Department of Chemistry and Biochemistry, Ludwig-Maximilians University Munich, Munich, Germany; University of Iowa, United States of America

## Abstract

Hereditary defects in the transcription-coupled nucleotide excision repair (TC-NER) pathway of damaged DNA cause severe neurodegenerative disease Cockayne syndrome (CS), however the origin and chemical nature of the underlying DNA damage had remained unknown. To find out, to which degree the structural properties of DNA lesions determine the extent of transcription arrest in human CS cells, we performed quantitative host cell reactivation analyses of expression vectors containing various synthetic adducts. We found that a single 3-(deoxyguanosin-*N* ^2^-yl)-2-acetylaminofluorene adduct (dG(*N* ^2^)-AAF) constitutes an unsurmountable obstacle to transcription in both CS-A and CS-B cells and is removed exclusively by the CSA- and CSB-dependent pathway. In contrast, contribution of the CS proteins to the removal of two other transcription-blocking DNA lesions – *N*-(deoxyguanosin-8-yl)-2-acetylaminofluorene (dG(C8)-AAF) and cyclobutane thymine-thymine (TT) dimer – is only minor (TT dimer) or none (dG(C8)-AAF). The unique properties of dG(*N* ^2^)-AAF identify this adduct as a prototype for a new class of DNA lesions that escape the alternative global genome repair and could be critical for the CS pathogenesis.

## Introduction

Cockayne syndrome (CS) is an incurable genetic disease characterized by severe neurological and developmental abnormalities, growth failure and pathological changes in multiple organs [1]. CS has been linked to a defect in the transcription-coupled nucleotide excision repair pathway (TC-NER), which is normally initiated by arrest of the elongating RNA polymerase II (RNAPII) complexes at bulky DNA adducts [2], [3]. As a result, CS patients generally manifest impaired recovery of transcription after DNA damage induction (such as UV-irradiation), which is a useful diagnostic criterion for CS [4]. Pathogenic mutations in CS were mapped to the genes *CSB/ERCC6* (about 60% of cases) and *CSA/ERCC8* [5]. In response to UV damage, both gene products regulate (de-)ubiquitination reactions which are essential for displacement of the stalled RNAPII [6] – CSB as one of the initial sensors of the damage-arrested RNAPII [7] and CSA as an interaction partner of the CRL4 E3 ubiquitin ligase complex [8], [9]. However, since organs deep in the body are not exposed to UV, the nature and origins of DNA damage responsible for CS remain speculative [10], [11].

If the TC-NER defect is crucial for the CS phenotype, there must exist a DNA lesion that would fulfill the following criteria: (i) a strong capacity to impede the RNAPII-driven transcription; (ii) requirement of both CSB and CSA proteins for efficient recovery of transcription in cells; and (iii) the lack of efficient alternative repair pathways in the absence of TC-NER. The capacity to arrest the elongating RNAPII has been demonstrated for a few DNA modifications, including – besides the UV-induced cyclobutane pyrimidine dimers and (6–4) photoproducts – the adducts of chemical carcinogens, such as N-2-acetylaminofluorene (AAF) [12], [13]. AAF forms two types of adducts with guanine bases – *N*-(deoxyguanosin-8-yl)-2-acetylaminofluorene (dG(C8)-AAF) and 3-(deoxyguanosin-*N* ^2^-yl)-2-acetylaminofluorene (dG(*N* ^2^)-AAF) – with remarkably different topologies of the modified DNA duplexes. The presence of dG(C8)-AAF in DNA results in a large degree of helical distortion [14] and significant thermodynamic destabilization of the duplex which facilitates the damage recognition by human nucleotide excision repair (NER) factors [15], [16]. In contrast, (dG(*N* ^2^)-AAF) is accommodated in the minor groove of the helix, increasing the thermodynamic stability of the duplex DNA [17]. According to the prevailing bipartite model of the damage recognition in NER [18], this is expected to impede the detection of dG(*N* ^2^)-AAF. Here, we tested whether the strikingly different thermodynamic properties of the two AAF adducts have implications for the repair pathway, which led us to finding that only dG(*N* ^2^)-AAF requires the CSA and CSB proteins.

## Materials and Methods

### Oligonucleotides

All synthetic deoxyribo-oligonucleotides were HPLC-purified and verified by MALDI-TOF mass spectrometry. The sequence was 5′-CATTGCTTCGCTAGCACG, where the underlined TT and G indicate the positions of the TT dimer and the acetylaminofluorene (AAF) adducts. Unmodified oligonucleotide was purchased from Eurofins MWG Operon (Ebersberg, Germany). Oligonucleotide containing TT dimer was from TriLink BioTechnologies (San Diego, CA). Oligonucleotides containing the *N*-(deoxyguanosin-8-yl)-2-acetylaminofluorene (dG(C8)-AAF) and 3-(deoxyguanosin-*N* ^2^-yl)-2-acetylaminofluorene (dG(*N* ^2^)-AAF) adducts were produced as following.

The dG(C8)-AAF phosphoramidite containing an isopropylphenoxyacetyl group at the *N* ^2^ position for solid phase DNA synthesis was prepared as previously published [19], [20] and incorporated into DNA using ultra mild conditions [21]. The dG(*N* ^2^)-AAF phosphoramidite for solid phase DNA synthesis was prepared as previously published by Johnson and coworkers and incorporated into DNA using standard conditions [22]. Deprotection and cleavage of the oligodeoxynucleotides from the CPG carrier containing dG(*N* ^2^)-AAF were carried out in a mixture of saturated (7 M) ammonia solution in water:ethanol (3∶1) at 17 °C overnight. Oligonucleotide synthesis was performed on an ABI 394 Nucleic Acid Synthesis System (Life Technologies, Darmstadt, Germany). Phosphoramidites for dA, dC, dG, dT and CPG carriers were obtained from Glen Research (Sterling, VA) or Link Technologies (Bellshill, Scotland). The coupling time for the modified phosphoramidites was extended to 2 × 7 min.

DNA purification was conducted on analytical and preparative HPLC (Waters, Eschborn, Germany) using Nucleodur or Nucleosil columns (250 × 4 mm, C18ec, particle size 3 μm or 250 × 10 mm, C18ec, particle size 5 μm) from Macherey-Nagel (Düren, Germany). The applied buffer was 0.1 M triethylammonium acetate in water and 0.1 M triethylammonium acetate in an 80% aqueous MeCN buffer system. The fractions were checked for purity by analytical HPLC. The purified oligonucleotides were concentrated using a Savant SpeedVac system (Thermo Scientific, Dreieich, Germany) and desalted with Sep-Pak cartridges (Waters).

### Construction of vectors containing single adducts

Mammalian expression vectors pZAJ-5w-AGC and pZAJ-5c-AGC, containing the 5′-CATTGCTTCGCTAGCACGCATTGC sequence in opposite orientations within the the 5′ untranslated region (5′-UTR) of a gene coding for the enhanced green fluorescent protein (EGFP), were described previously [23]. A pair of vectors, containing the same sequences in an arbitrarily chosen non-genic region upstream of the immediate early CMV promoter, was constructed by analogous procedures from the same maternal pZAJ vector.

Site-specific double nicks were produced in either the transcribed or coding DNA strand with the Nb.BsrDI endonuclease (NEB GmbH, Frankfurt am Main, Germany). After melting the excised 18-mers away, the synthetic oligonucleotides (unmodified or containing the specified adducts) were inserted and the nicks sealed by the protocol described previously [24]. The presence of dG(C8)-AAF and dG(*N* ^2^)-AAF in vector DNA was verified by the inhibition of generation of a double-stranded break by the NheI restriction endonuclease; the presence of TT dimer was verified by incision with T4 endonuclease V (**Figure S1**).

### Cell lines

Immortalised human skin fibroblasts from patients with mutations in the specified nucleotide excision repair genes were obtained from the NIGMS Human Genetic Cell Repository, Coriell Institute for Medical Research (Camden, New Jersey, USA). The CS-B cell line was CS1ANps3g2 (GM16095); the CS-A cell line was CS3BEs3gl (GM16094); the XP-A cell line was XP20S (GM04312); and the XP-C cell line was XP4PA-SV-EB (GM15983). The matched repair-proficient cell line was MRC-5 VA1 (AG10076). The CSB-corrected cell line was CS1ANps3g2 transfected with the CSB cDNA expression construct [25], kindly provided by Kiyoji Tanaka (Osaka University, Japan).

### CSB protein knockdown

Stable knockdown of the *CSB* gene in HeLa cells was achieved exactly as described previously for another gene [23]. For shRNA targeting of the *CSB* gene, the specific complimentary oligonucleotides containing the BglII- and HindIII-compatible overhangs were annealed and cloned into the pENTR/pSuper+ vector (Addgene, Cambridge, MA) downstream of the H1 promoter. The sequences of oligonucleotides were the following: 5′-GATCCCCGGAAGAAGCAAGGTTGTAATTCAAGAGATTACAACCTTGCTTCTTCCTTTTTGGAAA (forward) and 5′- AGCTTTTCCAAAAAGGAAGAAGCAAGGTTGTAATCTCTTGAATTACAACCTTGCTTCTTCCGGG (reverse). The CSB protein levels in individual clones were monitored by Western blotting with the A301-345A rabbit polyclonal antibody (Bethyl Laboratories, Inc., Montgomery, TX) raised against the amino acid residues 1–50 of human CSB.

### Transfections and gene expression analyses

Exponentially growing cells were co-transfected with equal amounts of one of the EGFP-encoding vectors with an inserted synthetic oligonucleotide (either unmodified or containing the specified adducts) and pDsRed-Monomer-N1 vector (Clontech, Saint-Germain-en-Laye, France). Transfections were performed with the help of Effectene (QIAGEN, Hilden, Germany). Cells were fixed 24 hours post transfections, and EGFP expression was measured in individual cells by flow cytometry, as described in detail previously [26].

## Results

### A unique dG(*N^2^*)-AAF abolishes gene expression in CS cells

**Figure 1:**
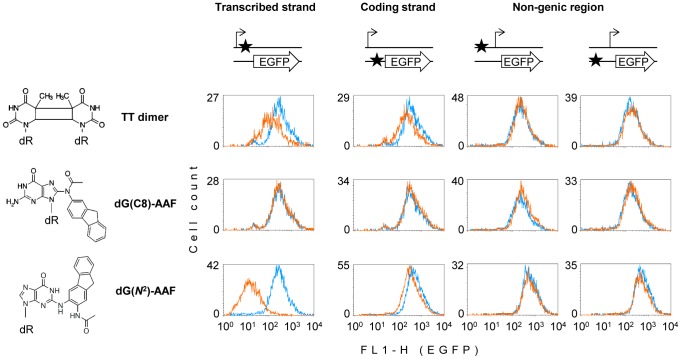
Effects of single adducts on the EGFP gene expression in the GM16095 (CS-B) cell line. The protein coding sequence (arrow) and the transcription start (broken arrow) are represented out of scale, along with the adduct position (star) with respect to the transcription unit. Overlaid fluorescence distribution plots were obtained by flow cytometric analyses of cells transfected with vectors carrying the specified adducts (amber line) and with the control constructs obtained by incorporation of the unmodified deoxyribo-oligonucleotide (blue line).

Our strategy was to construct mammalian expression vectors carrying unique synthetic DNA base modifications in defined positions of the reporter EGFP gene [24], followed by quantitative gene expression analyses in human host cells carrying mutations in the *CSA* and *CSB* genes. We produced synthetic oligonucleotides containing unique dG(C8)-AAF and dG(*N* ^2^)-AAF adducts and inserted them into the specially designed expression vectors, in various positions (**Figure 1** **, Figure S1**). Analogously constructed vectors containing a single cyclobutane thymine-thymine (TT) dimer (**Figure 1** **, Figure S1**) were used for comparison. In a human CS-B cell line, we detected a potent inhibition of the gene expression by dG(*N* ^2^)-AAF in the transcribed DNA strand of the EGFP gene, whereas the construct containing dG(C8)-AAF in the same position was expressed at the same level as control unmodified DNA (**Figure 1**). These results indicate that dG(C8)-AAF is efficiently removed by a CSB-independent repair pathway, which is in line with a previous report [27]. In contrast, dG(*N* ^2^)-AAF can be neither removed nor bypassed in the absence of the CSB protein, constituting an unconditional roadblock for the RNAPII transcription. It is interesting to note that the TT dimer, which is a widely accepted “golden standard” for a transcription-blocking lesion and a classical substrate for TC-NER, exhibited a much milder grade of inhibition of the gene expression than dG(*N* ^2^)-AAF. We interpret this attenuated effect as a result of removal of a significant fraction of TT dimers by global genome NER (GG-NER) – an alternative (CSA-, CSB- and transcription-independent) pathway initiated by the specific damage recognition factors XPC-RAD23B and UV-DDB [3], [28].

To test whether the inhibitory effect of the dG(*N* ^2^)-AAF adduct on the gene expression requires the damage presence within the transcribed DNA strand, we also analyzed the effects of the same three adducts positioned in the opposite DNA strand and in a non-transcribed sequence upstream of the promoter. Only minimal effects were caused by dG(*N* ^2^)-AAF and TT dimers in the coding strand of the gene and none of the adducts could affect the EGFP expression when located in the upstream non-transcribed region (**Figure 1**), thus indicating that a direct interaction of transcription complexes with the dG(*N* ^2^)-AAF adduct is necessary for the inhibition of the gene expression.

### CSB is required for removal of the transcription-blocking dG(*N^2^*)-AAF

**Figure 2:**
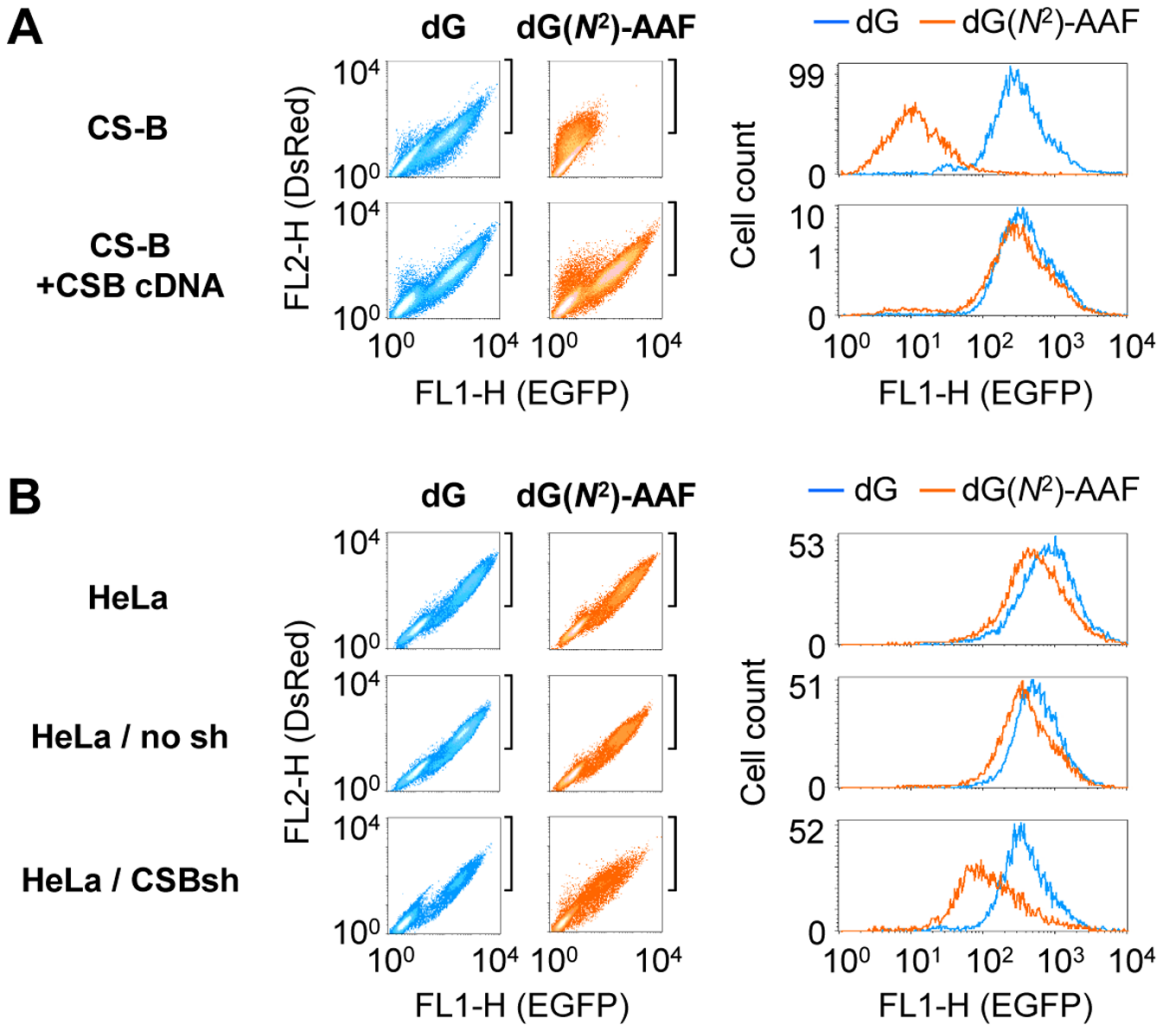
Modulation of the effect of dG(*N^2^*)-AAF by CSB. Host cell reactivation of the EGFP expression in pairs of isogenic cell lines with different CSB expression statuses. Cells were transfected with constructs containing a unique dG(*N* ^2^)-AAF (amber colors) or dG (blue colors) in the transcribed strand of the EGFP gene. Cells were selected according to the expression of DsRed as a transfection marker (the gated region shown by square brackets near the dot density plots) to generate the overlaid fluorescence distribution plots (on the right). (A) The effect of complementation of the CSB deficiency by expression of the CSB cDNA. (B) The effect of CSB knockdown by expression of the specific shRNA (CSBsh). See also Figures S3 and S4.

To test whether the *CSB* gene defect is causal for the inhibitory effect of single dG(*N* ^2^)-AAF adduct on the gene transcription, we transfected the same constructs in parallel into the CS-B cell line (GM16095) and the isogenic cell line stably expressing the CSB cDNA [25]. The EGFP expression was restored almost completely in the CSB-complemented cells (**Figure 2A** **, Figure S2**), indicating that a functional *CSB* gene is required for the efficient recovery of transcription. To test the effect of CSB in a different genetic background, we stably knocked down CSB expression in HeLa cells by permanent transfection with the specific short hairpin (sh) RNA. This resulted in an approximately 70% reduction of the CSB protein level (**Figure S3**), which in turn hindered transcription of the dG(*N* ^2^)-AAF-containing expression construct (**Figure 2A** **, Figure S4**), Altogether, the results indicate that the requirement for CSB is independent from the genetic background.

### TC-NER is indispensable for removal of the transcription-blocking dG(*N^2^*)-AAF

**Figure 3:**
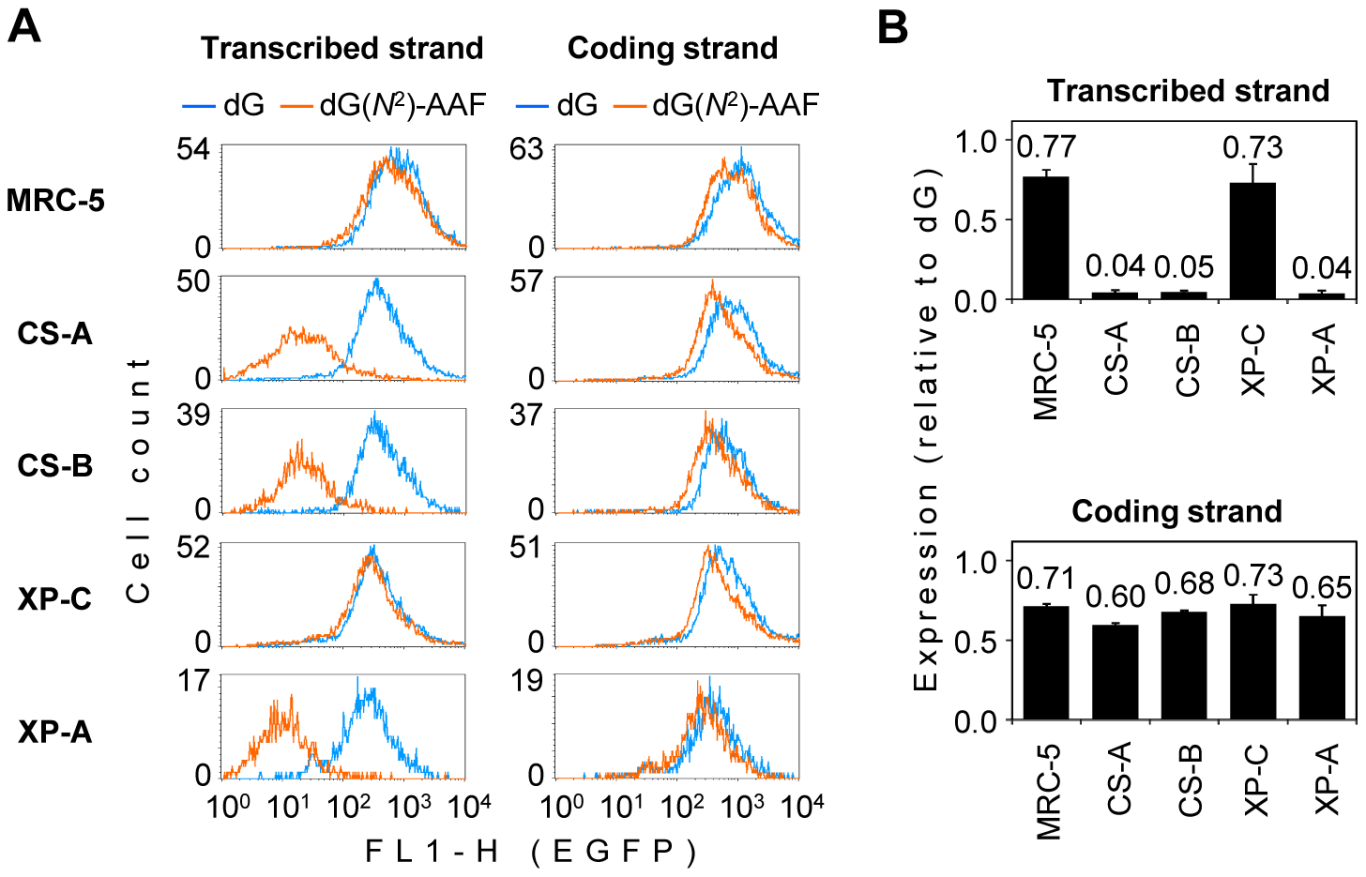
Exclusive requirement of TC-NER for the removal of transcription-blocking dG(*N^2^*)-AAF. Host cell reactivation in human cell lines of the specified nucleotide excision repair complementation groups (CS-A, CS-B, XP-C and XP-A) and in the repair proficient cell line (MRC-5). The expression constructs contained a unique dG(*N* ^2^)-AAF or dG in the specified DNA strand. (A) Overlaid fluorescence distribution plots obtained in a representative experiment. All cell lines were transfected in parallel with the same constructs. (B) Mean relative expression (dG(*N* ^2^)-AAF/dG) calculated for multiple transfections with independent preparations of the expression constructs (n = 3, +/− s.d.).

To obtain further information about the relative contributions of TC-NER and GG-NER to the repair of dG(*N* ^2^)-AAF, the expression was analyzed in parallel in the CS-A and CS-B cell lines (deficient in the TC-NER), an XP-C cell line (deficient in the GG-NER), and an XP-A cell line (deficient in both the TC-NER and GG-NER pathways). A fully repair proficient MRC-5 cell line was used for comparison. In the XP-C cell line, the expression of the construct containing dG(*N* ^2^)-AAF in the transcribed DNA strand was as good as in the control MRC-5 cells, indicating that the GG-NER defect does not compromise the efficiency of the adduct removal (**Figure 3** **, Figure S5**). In the CS-A and CS-B cell lines, the strength of the negative effect of the dG(*N* ^2^)-AAF adduct on the gene expression was quantitatively the same as in the XP-A cell line. Since the TC-NER defect was phenotypically equivalent to the total absence of NER, we conclude that dG(*N* ^2^)-AAF in the transcribed DNA strand can only be repaired by the transcription-coupled pathway.

### Host cell reactivation efficiencies of vectors carrying various adducts in CS-A cells

**Figure 4:**
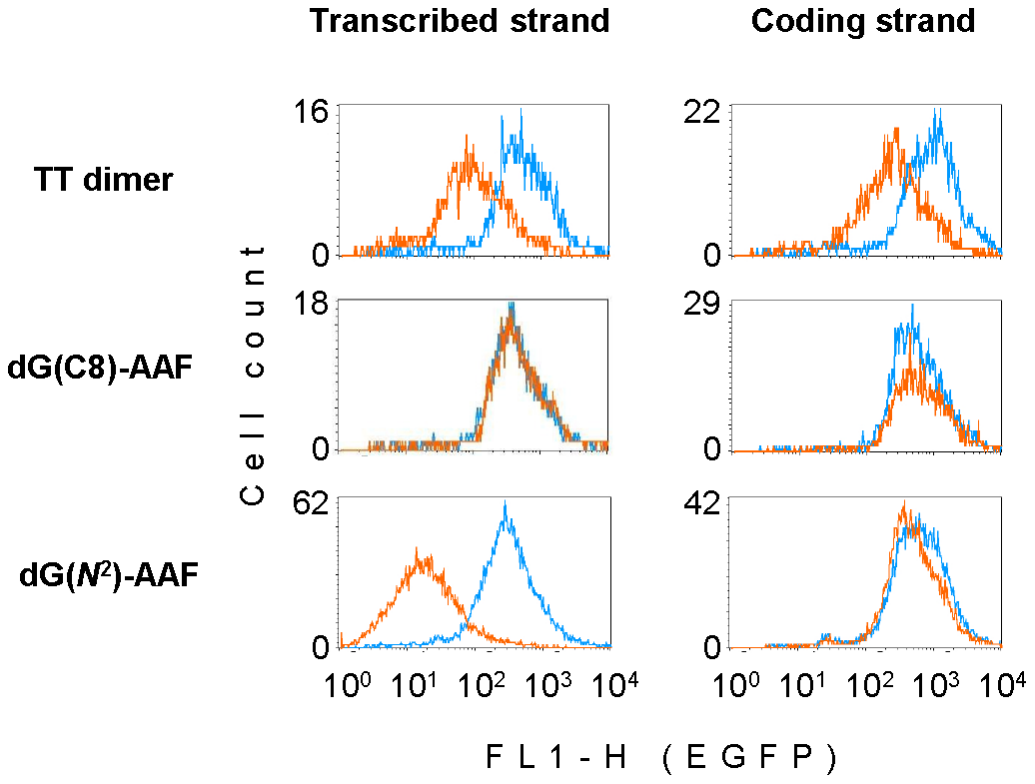
Effects of single adducts on the EGFP gene expression in the GM16094 (CS-A) cell line. Overlaid fluorescence distribution plots of cells transfected with vectors carrying the specified adducts (amber line) and with the control constructs obtained by incorporation of the unmodified deoxyribo-oligonucleotide (blue line).

Data shown in **Figure 3** indicate that the products of *CSA* and *CSB* genes are of the same critical importance for removal of the transcription-blocking dG(*N* ^2^)-AAF in cells. We questioned how harmful are two other bulky DNA lesions – dG(C8)-AAF and TT dimer – for the gene expression in CS-A cells. Regardless of the DNA strand concerned, the constructs containing dG(C8)-AAF and the corresponding adduct-free control vectors were expressed at the same levels (**Figure 4**), exactly recapitulating the results previously obtained in the CS-B cell line (**Figure 1**). TT dimer induced inhibition of the gene expression which was milder than in the case of dG(*N* ^2^)-AAF and exhibited a less pronounced DNA strand-specificity, which may indicate some indirect interference of the lesion with transcription (**Figure 4**). Both findings resembled the effects previously observed in the CS-B cell line (**Figure 1**). Altogether, of the three lesions investigated, dG(*N* ^2^)-AAF turned out to be most critical for transcription both in CS-A and CS-B cells, if present in the transcribed DNA strand. None of the lesions could influence the EGFP expression when located in the upstream non-transcribed region (data not shown).

## Discussion

In summary, of the three bona fide transcription blocking DNA base modifications investigated here, only dG(*N* ^2^)-AAF constitutes an insuperable transcription block in human CS cells. Two other DNA lesions are repaired or bypassed to a significant (TT dimer) or full (dG(C8)-AAF) extent. The removal of dG(*N* ^2^)-AAF from the transcribed DNA strand of the actively transcribed gene takes place exclusively by the TC-NER pathway which requires both CSA and CSB proteins, whereas the GG-NER pathway (initiated by the XPC gene product) is unimportant. This finding explains the remarkably long (>11 months) persistence time of dG(*N* ^2^)-AAF in rats fed with 2-nitrofluorene [29]. In a striking contrast, the contribution of TC-NER to the removal of another AAF adduct (dG(C8)-AAF) from the same transcribed gene is negligible, providing a reason for the absence of a strand-selective repair of dG(C8)-AAF in the endogenous *ADA* gene [27].

Comparison between the two AAF adducts suggests that GG-NER may be generally inefficient for adducts which have minimal influence on the DNA duplex structure and thermodynamic stability, leaving TC-NER as the only possible repair pathway. Such DNA lesions must be especially harmful in the CS-A and CS-B patients. Thereby, dG(*N* ^2^)-AAF can be regarded as a close prototype for unknown DNA lesion(s) that could be responsible for the molecular pathology of CS. Adducts with similar structural and thermodynamic features can be formed by active metabolites of environmental and dietary compounds (such as arylamines and heterocyclic amines), raising a possibility that manifestation of the clinical features of CS can be influenced by environmental exposures and the individual metabolic phenotype. This would explain the lack of good correlation between the patients' genotype and the severity of the disease [5], including the absence of the characteristic CS phenotype in a few described cases [30], [31].

## Supporting Information

Figure S1 **Verification of the incorporation of oligonucleotides containing the specified adducts into vector DNA.** The presence of acetylaminofluorene adducts (dG(C8)-AAF or dG(*N* ^2^)-AAF, indicated by the asterisk in the DNA sequence) within the unique NheI cleavage sequence (5′-GCTAGC) was verified by inhibition of the NheI endonuclease activity (left panel). The incorporation of thymine dimer was verified by incision with T4 endonuclease V (T4 EV) which cleaves the *N*-glycosylic bond of the 5′ thymine of the dimer and the phosphodiester bond 3′ to the resulting abasic site (right panel). Images show typical vector preparations analyzed in agarose gels containing ethidium bromide.
(PDF)

Figure S2 **Host cell reactivation of the EGFP expression in the CS-B cell line and the isogenic cell line corrected by expression of the CSB cDNA.** Transfected constructs contained a unique dG(*N* ^2^)-AAF in either the transcribed or non-transcribed (coding) strand of the EGFP gene, as indicated. Extended data for the experiment shown in Figure 2a.
(PDF)

Figure S3 **Stable knockdown of the endogenous CSB expression in HeLa cells.** Single clones were selected following transfections with empty vector (no sh) or the vector expressing the shRNA designed to target the *CSB* gene (CSBsh, three different clones) and analyzed by Western blot. Of the two bands recognized by the CSB antibody, one corresponds to the full-length CSB protein (arrow). This band is absent in the extracts obtained from the CS-B cell line (GM16095).
(PDF)

Figure S4 **Host cell reactivation of the EGFP expression in HeLa cells and the derived cell lines with different CSB expression statuses.** Clonal cell lines stably transfected with empty vector (no sh) or the vector expressing the CSB-specific shRNA (CSBsh, clone 21) were transfected with constructs containing a unique dG(*N* ^2^)-AAF in either the transcribed or non-transcribed (coding) strand of the EGFP gene, as indicated. Extended data for the experiment shown in Figure 2b.
(PDF)

Figure S5 **Host cell reactivation in human cell lines of the specified nucleotide excision repair complementation groups (CS-A, CS-B, XP-C and XP-A) and in the repair proficient cell line (MRC-5).** The expression constructs contained synthetic oligonucleotides with a unique dG(*N* ^2^)-AAF (amber colors) or dG (blue colors) in the specified DNA strand. Dot density plots obtained in a representative experiment and the corresponding overlaid fluorescence distribution plots. Extended data for the experiment shown in Figure 3.
(PDF)
