# Supplementary material for: Cockayne Syndrome: Varied Requirement of Transcription-Coupled Nucleotide Excision Repair for the Removal of Three Structurally Different Adducts from Transcribed DNA
Source: PLoS One. 2014 Apr 8;9(4):e94405. doi: 10.1371/journal.pone.0094405 (PMC3979923; doi:10.1371/journal.pone.0094405)
Supplement: Figure S1 — Verification of the incorporation of oligonucleotides containing the specified adducts into vector DNA. The presence of acetylaminofluorene adducts (dG(C8)-AAF or dG(N 2)-AAF, indicated by the asterisk in the DNA sequence) within the unique NheI cleavage sequence (5′-GCTAGC) was verified by inhibition of the NheI endonuclease activity (left panel). The incorporation of thymine dimer was verified by incision with T4 endonuclease V (T4 EV) which cleaves the N-glycosylic bond of the 5′ thymine of the dimer and the phosphodiester bond 3′ to the resulting abasic site (right panel). Images show typical vector preparations analyzed in agarose gels containing ethidium bromide. (PDF) [file pone.0094405.s001.pdf]

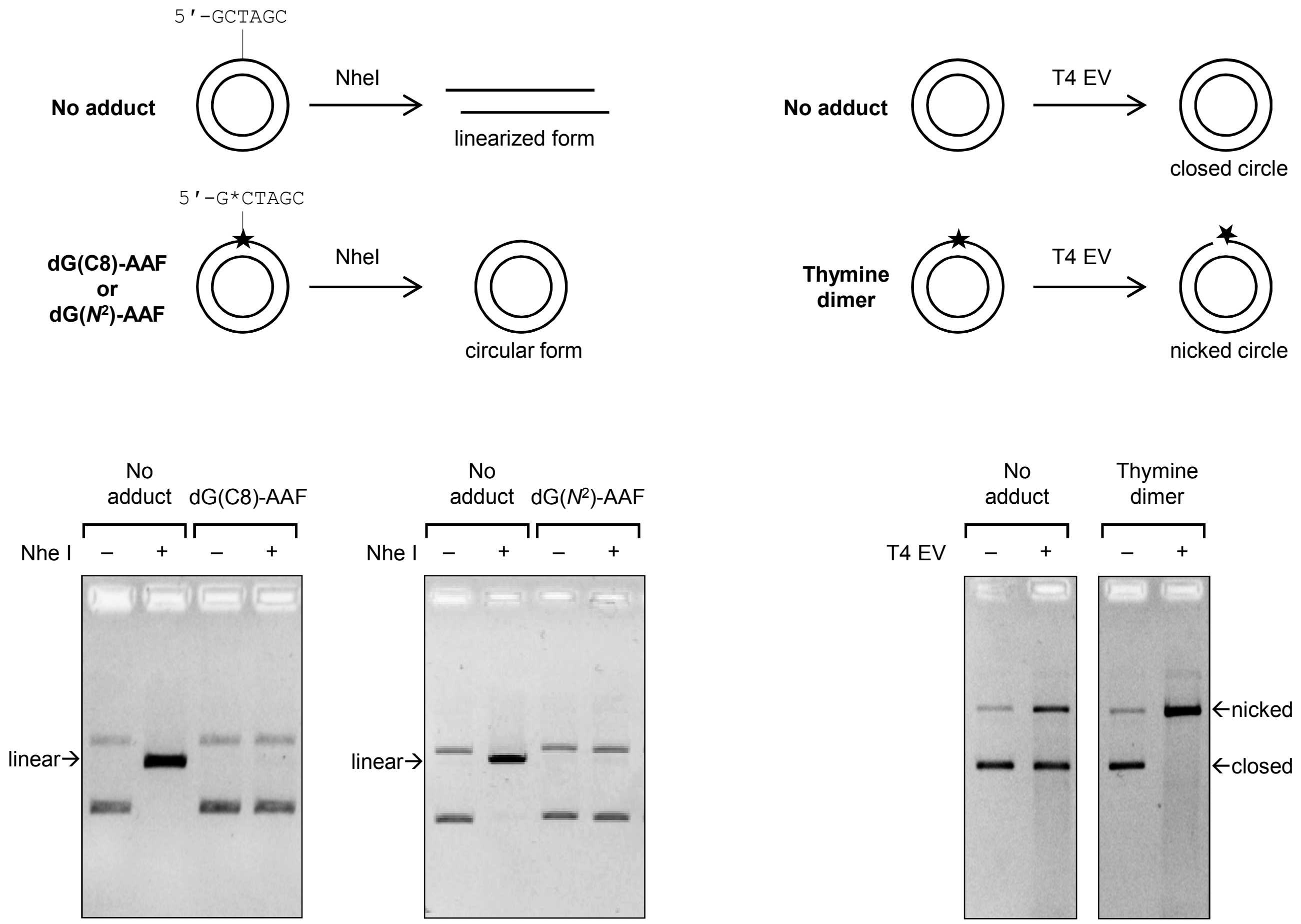

Supporting Figure 1. Verification of the incorporation of oligonucleotides containing the specified adducts into vector DNA. The presence of acetylaminofluorene adducts (dG(C8)-AAF or dG(N^2)-AAF, indicated by the asterisk in the DNA sequence) within the unique NheI cleavage sequence (5′-GCTAGC) was verified by inhibition of the NheI endonuclease activity (left panel). The incorporation of thymine dimer was verified by incision with T4 endonuclease V (T4 EV) which cleaves the *N*-glycosylic bond of the 5′ thymine of the dimer and the phosphodiester bond 3′ to the resulting abasic site (right panel). Images show typical vector preparations analyzed in agarose gels containing ethidium bromide.
