# Supplementary material for: Cockayne Syndrome: Varied Requirement of Transcription-Coupled Nucleotide Excision Repair for the Removal of Three Structurally Different Adducts from Transcribed DNA
Source: PLoS One. 2014 Apr 8;9(4):e94405. doi: 10.1371/journal.pone.0094405 (PMC3979923; doi:10.1371/journal.pone.0094405)
Supplement: Figure S2 — Host cell reactivation of the EGFP expression in the CS-B cell line and the isogenic cell line corrected by expression of the CSB cDNA. Transfected constructs contained a unique dG(N 2)-AAF in either the transcribed or non-transcribed (coding) strand of the EGFP gene, as indicated. Extended data for the experiment shown in Figure 2a. (PDF) [file pone.0094405.s002.pdf]

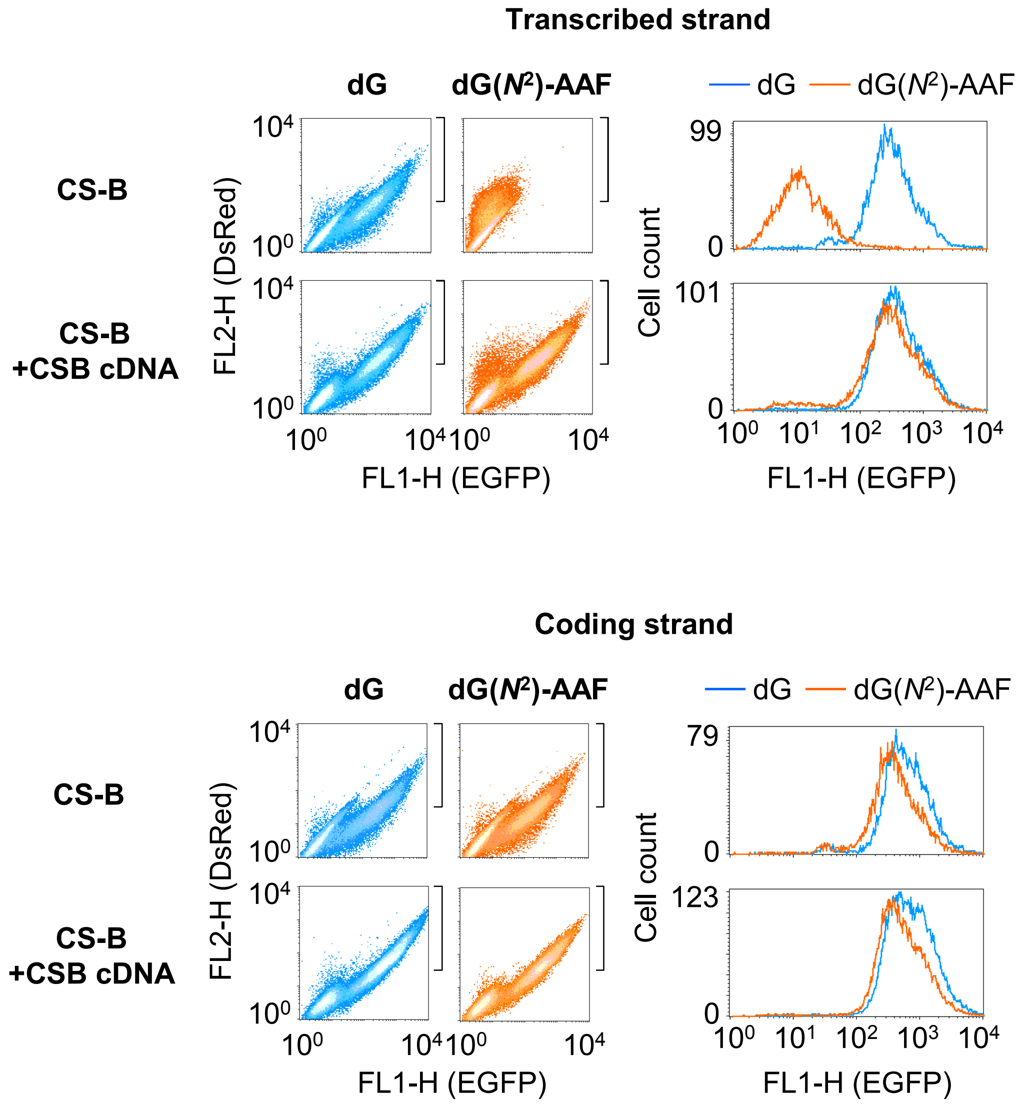

Supporting Figure 2. Host cell reactivation of the EGFP expression in the CS-B cell line and the isogenic cell line corrected by expression of the CSB cDNA. Transfected constructs contained a unique dG(N^2)-AAF in either the transcribed or non-transcribed (coding) strand of the EGFP gene, as indicated. Extended data for the experiment shown in Figure 2a.
