# Supplementary material for: Cockayne Syndrome: Varied Requirement of Transcription-Coupled Nucleotide Excision Repair for the Removal of Three Structurally Different Adducts from Transcribed DNA
Source: PLoS One. 2014 Apr 8;9(4):e94405. doi: 10.1371/journal.pone.0094405 (PMC3979923; doi:10.1371/journal.pone.0094405)
Supplement: Figure S3 — Stable knockdown of the endogenous CSB expression in HeLa cells. Single clones were selected following transfections with empty vector (no sh) or the vector expressing the shRNA designed to target the CSB gene (CSBsh, three different clones) and analyzed by Western blot. Of the two bands recognized by the CSB antibody, one corresponds to the full-length CSB protein (arrow). This band is absent in the extracts obtained from the CS-B cell line (GM16095). (PDF) [file pone.0094405.s003.pdf]

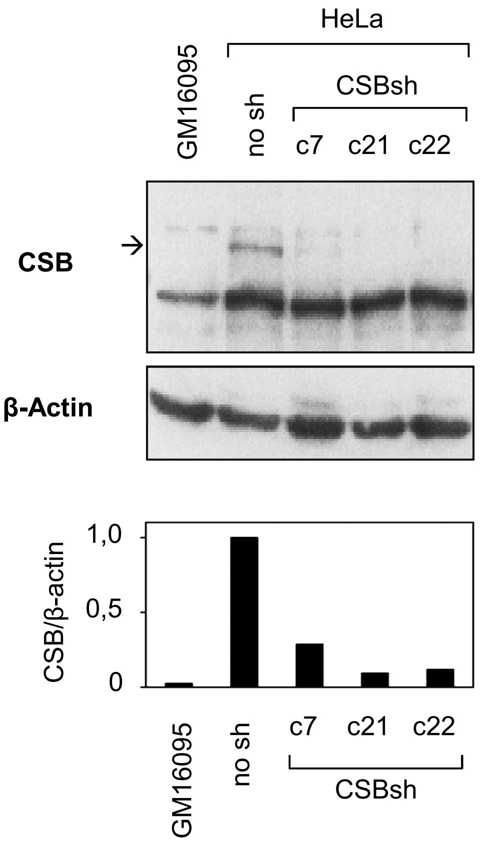

Supporting Figure 3. Stable knockdown of the endogenous CSB expression in HeLa cells. Single clones were selected following transfections with empty vector (no sh) or the vector expressing the shRNA designed to target the *CSB* gene (CSBsh, three different clones) and analyzed by Western blot. Of the two bands recognized by the CSB antibody, one corresponds to the full-length CSB protein (arrow). This band is absent in the extracts obtained from the CS-B cell line (GM16095).
