# Supplementary material for: Cockayne Syndrome: Varied Requirement of Transcription-Coupled Nucleotide Excision Repair for the Removal of Three Structurally Different Adducts from Transcribed DNA
Source: PLoS One. 2014 Apr 8;9(4):e94405. doi: 10.1371/journal.pone.0094405 (PMC3979923; doi:10.1371/journal.pone.0094405)
Supplement: Figure S4 — Host cell reactivation of the EGFP expression in HeLa cells and the derived cell lines with different CSB expression statuses. Clonal cell lines stably transfected with empty vector (no sh) or the vector expressing the CSB-specific shRNA (CSBsh, clone 21) were transfected with constructs containing a unique dG(N 2)-AAF in either the transcribed or non-transcribed (coding) strand of the EGFP gene, as indicated. Extended data for the experiment shown in Figure 2b. (PDF) [file pone.0094405.s004.pdf]

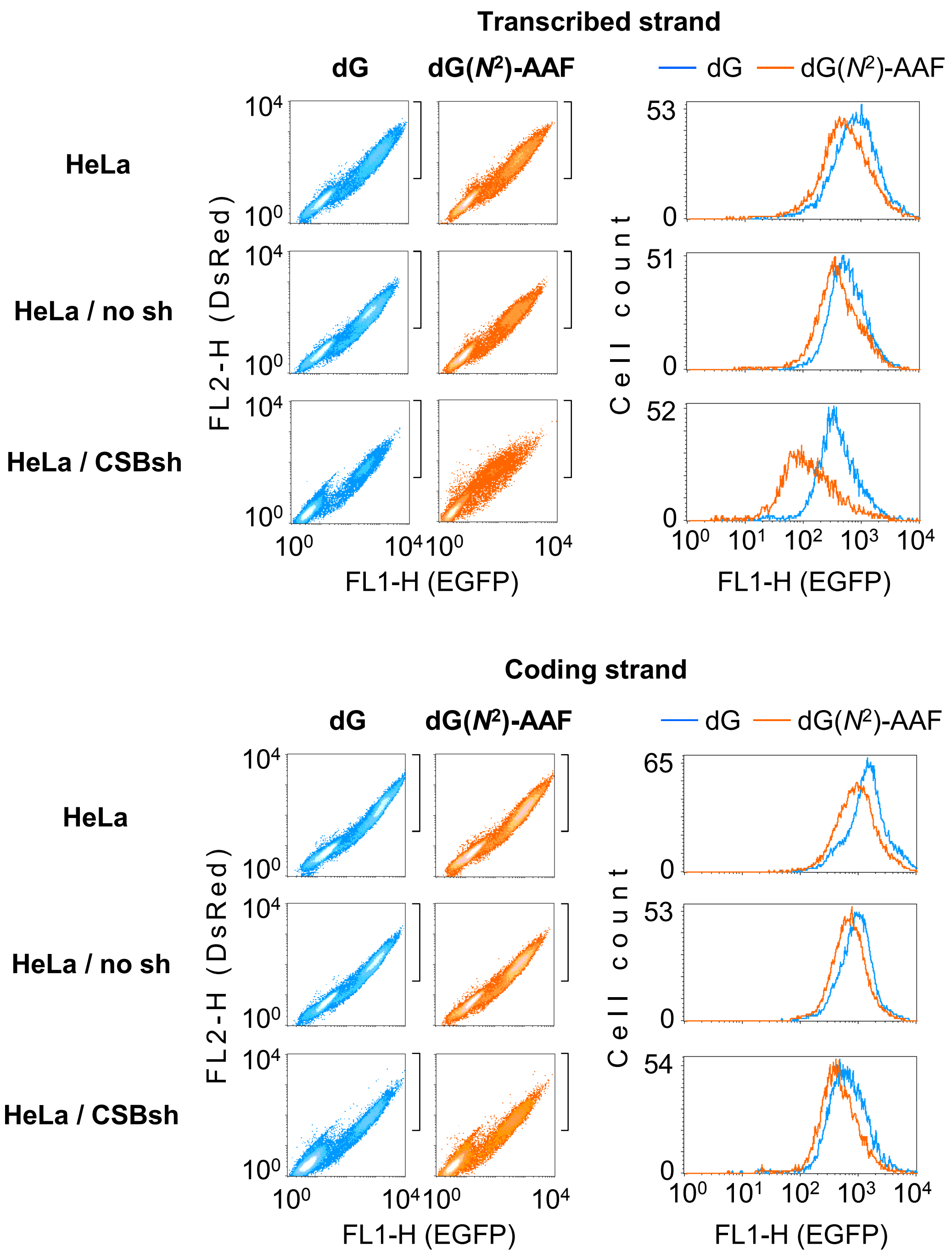

Supporting Figure 4. Host cell reactivation of the EGFP expression in HeLa cells and the derived cell lines with different CSB expression statuses. Clonal cell lines stably transfected with empty vector (no sh) or the vector expressing the CSB-specific shRNA (CSBsh, clone 21) were transfected with constructs containing a unique dG(N^2)-AAF in either the transcribed or non-transcribed (coding) strand of the EGFP gene, as indicated. Extended data for the experiment shown in Figure 2b.
