# Supplementary material for: Cockayne Syndrome: Varied Requirement of Transcription-Coupled Nucleotide Excision Repair for the Removal of Three Structurally Different Adducts from Transcribed DNA
Source: PLoS One. 2014 Apr 8;9(4):e94405. doi: 10.1371/journal.pone.0094405 (PMC3979923; doi:10.1371/journal.pone.0094405)
Supplement: Figure S5 — Host cell reactivation in human cell lines of the specified nucleotide excision repair complementation groups (CS-A, CS-B, XP-C and XP-A) and in the repair proficient cell line (MRC-5). The expression constructs contained synthetic oligonucleotides with a unique dG(N 2)-AAF (amber colors) or dG (blue colors) in the specified DNA strand. Dot density plots obtained in a representative experiment and the corresponding overlaid fluorescence distribution plots. Extended data for the experiment shown in Figure 3. (PDF) [file pone.0094405.s005.pdf]

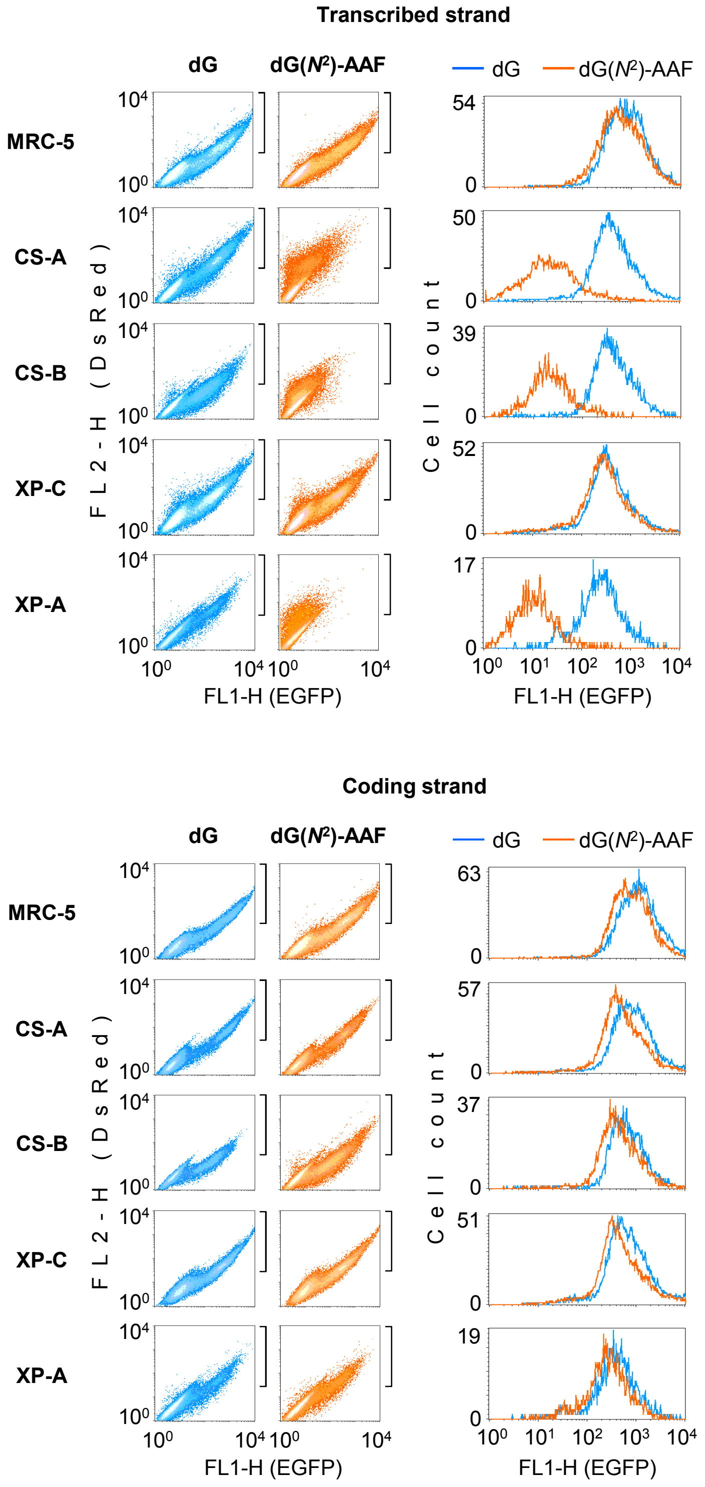

Supporting Figure 5. Host cell reactivation in human cell lines of the specified nucleotide excision repair complementation groups (CS-A, CS-B, XP-C and XP-A) and in the repair proficient cell line (MRC-5). The expression constructs contained synthetic oligonucleotides with a unique dG(N^2)-AAF (amber colors) or dG (blue colors) in the specified DNA strand. Dot density plots obtained in a representative experiment and the corresponding overlaid fluorescence distribution plots. Extended data for the experiment shown in Figure 3.
